# Development of a comprehensive dietary dataset for idiopathic granulomatous mastitis (IGM) patients and matched controls: protocol, implementation, and future directions for nutrition-based research

**DOI:** 10.1186/s13104-025-07507-6

**Published:** 2025-10-22

**Authors:** Sadaf Alipour, Sakineh Shab-Bidar, Bita Eslami, Ramesh Omranipour, Shiller HessamiAzar, Marzieh Orouji, Ramin Mansouri, Sara Alipour, Kiana Kimiaei-Asadi, Reyhaneh Aghajani

**Affiliations:** 1 Breast Diseases Research Center, Cancer Institute, Tehran University of Medical Sciences, Tehran, Iran; 2 Department of Surgery, Arash Women’s Hospital, Faculty of Medicine, Tehran University of Medical Sciences, Tehran, Iran; 3 Department of Community Nutrition, School of Nutritional Sciences and Dietetics, Tehran University of Medical Sciences, Tehran, Iran; 4 Department of Cancer Surgery, Cancer Institute, Faculty of medicine, Tehran University of Medical Sciences, Tehran, Iran; 5 Department of Nursing, Arash Women’s Hospital, Tehran University of Medical Sciences, Tehran, Iran; 6 School of Medicine, Student Research Committee, Hamadan University of Medical Sciences, Hamadan, Iran; 7 Cancer Institute, Imam Khomeini Complex Hospital, 2nd floor, Sadaf Building, Keshavraz Blvd, Tehran, 1419733141 Iran

## Abstract

**Objective:**

Idiopathic granulomatous mastitis (IGM) is a rare chronic inflammatory disease with unclear etiology and substantial morbidity. While immunological, hormonal, and infectious triggers have been proposed, the role of dietary factors in IGM pathogenesis remains underexplored despite emerging associations between diet and inflammatory conditions. This study describes the development of a comprehensive dataset of dietary intake and nondietary variables from IGM patients and matched controls, enabling future research into potential diet–IGM associations.

**Results:**

Between 2022 and 2024, a multicenter case‒control study enrolled 608 female patients with histologically confirmed IGM and 568 matched controls. Data included demographic, reproductive, and anthropometric measurements, hormonal profiles, and detailed dietary intake data evaluated via a validated 147-item food frequency questionnaire (FFQ). Nutrient intake was calculated using Nutritionist IV software with traditional Iranian foods. Variables were classified by importance, and strict criteria ensured data quality. Participants were from 36 cities across Iran. A total of 430,572 individual data points were generated, resulting in a comprehensive dataset encompassing dietary patterns alongside clinical and health-related variables. This dataset provides a critical foundation for future research on dietary factors in IGM onset, progression, and clinical management, supports evidence-based recommendations, and may guide standardized dietary guidelines for IGM patients.

**Supplementary Information:**

The online version contains supplementary material available at 10.1186/s13104-025-07507-6.

## Introduction

Idiopathic granulomatous mastitis (IGM) is a chronic inflammatory disease of the breast that was recognized in the 1970s [1], but many uncertainties remain regarding its etiology, triggering factors, and treatment. IGM is rare in most parts of the world but is less common in some areas, such as Turkey, Iran, China, India, and the Hispanic population of the USA [2].

In addition to its protracted course and challenges in long-term management, the disease frequently imposes significant morbidity, manifesting as disfiguring breast lesions and persistent fistulas. The hypotheses behind the cause of the disease are several, but immunological alterations, infectious causes, and hormonal triggers are the most likely [3]. While aspects of the reproductive life of a woman, as well as underlying diseases or exogenous hazards such as smoking, have been frequently explored [3], abnormalities arising secondary to breastfeeding problems and milk stasis have recently been highlighted as the main initiating mechanism of the disease [4–6] Nevertheless, even in this scenario, stasis may lead to leakage into breast tissue, initiating a cascade of immunologic or autoimmune reactions that cause tissue inflammation [7, 8].

However, dietary factors have been largely overlooked. Although tissue inflammation and heightened immune responses are associated with nutrient intake [9, 10], research specifically examining these links in idiopathic granulomatous mastitis (IGM) remains limited. Existing studies have focused primarily on isolated dietary components, often based on anecdotal observations. For example, while one study suggested a connection between spicy food consumption and IGM [11], another disputes this association [4]. Additionally, recent findings implicate food allergies in IGM pathogenesis [12]. Furthermore, differences in gut microbiota metabolites among IGM patients [13] suggest a potential dietary influence.

There is a substantial knowledge gap regarding the role of diet in IGM. To address this, we developed a comprehensive dataset of systematically collected dietary information from both IGM patients and a control group. This article outlines the dataset’s development protocol and highlights its key features.

## Methods

### Study design, settings, and participants

This project was approved by the Ethics Committee of Tehran University of Medical Sciences, Tehran, Iran (Ethics Code: IT.TUMS.IKHC.REC.1300.437). This was a multicenter case‒control study centered at the Breast Diseases Research Center (BDRC), Cancer Institute, TUMS, and was carried out from November 2022 to October 2024.

The case group (IGM Group, IGG) comprised patients from breast and IGM clinics across Iran. The control group (CG) included accompanying women at these clinics, as well as women visiting dermatology, infectious disease, and pediatric clinics (mothers of sick children). The rationale behind selecting these specific clinics was that these individuals belonged to the same healthcare settings as the cases, ensuring similar geographic, cultural, and socioeconomic backgrounds. They were accessible and were less likely to harbor diseases that had dietary concerns. Although medical history and other issues regarding eligibility criteria were asked from every individual before inclusion, we targeted a population that were more likely to fulfil the criteria. Participants in CG were matched to ICG patients on age, sex (all female), and place of living.

The IGM group (IGG) included female patients aged > 18 years with histologically confirmed IGM diagnosed within the past 6 months. The control group (CG) comprised women with no personal or first-degree family history of IGM. The exclusion criteria for both groups were liver/heart failure, diabetes, memory disorders, recent significant dietary changes (e.g., adoption of vegetarianism), a history of breast cancer, and current pregnancy or lactation.

### Data collection

Personal and demographic information, as well as data about reproductive and past medical history, were collected through interviews conducted by trained staff, and anthropometric variables were measured for all participants. The Persian language-validated version of a comprehensive 147-item food frequency questionnaire (FFQ) [14] was completed in detail. Previous studies employing this FFQ have demonstrated acceptable validity and reliability [14]. It represents every food item as a predefined standard portion size, and the time interval scales are presented as daily, weekly, monthly, or yearly consumption. The participants in the two groups were asked to report their consumption frequency for each item based on their intake patterns during the last 12 months. The English version of the 147-item FFQ is shown in Additional File 1.

An electronic online version of both questionnaires (diet and non-diet data) was developed to facilitate the interviews and enhance precision. The interviewers completed the online questionnaire, and the output was directly received at the project base (BDRC).

After completion of the FFQs, reported consumption amounts for each food item were converted to grams via standardized measurement guides [15]. Nutrient intake values were then calculated for each participant via Nutritionist IV software (First Databank, San Bruno, CA, USA), which utilizes the USDA Food Composition Database supplemented with traditional Iranian food items where applicable [16].

All the collected data together with the results of the nutrient value computations were entered into a statistical database platform specifically designed for this study. To verify the accuracy of data entry, a randomly chosen 10% of the data entered in each group were rechecked with their original forms and pertinent calculations.

## Results

The participants were recruited from 36 cities in Iran. After the exclusion of 10 patients with IGG due to incomplete data, 608 and 568 participants were ultimately included in the IGG and CG, respectively. The list of nondiet variables of the dataset is shown in Table 1.

**Table 1:** Nondiet variables recorded in the dataset for participants in the two groups

Row	Variables	IGM group	Control group	Row	Variables	IGM group	Control group
*A*	*Demographic*			*C*	*Anthropometrics*		
1	Patient age	✳	✳	17	Weight	✳	✳
2	City of birth	✳	✳	18	Height	✳	✳
3	Province of birth	✳	✳	19	Neck circumference^**^	✳	✳
4	City of residence	✳	✳	20	Waist circumference^**^	✳	✳
5	Province of residence	✳	✳	21	Hip circumference^**^	✳	✳
6	Education	✳	✳	**D**	**Hormone use**		
7	Job	✳	✳	22	Hx of OCP use	✳	✳
*B*	*Reproductive*			23	Duration of OCP use	✳	✳
8	Age at menarche	✳	✳	24	Last OCP use	✳	✳
9	Gravidity	✳	✳	25	Hx of HRT	✳	✳
10	Parity	✳	✳	26	Duration of HRT	✳	✳
11	Abortion Hx	✳	✳	27	Last HRT	✳	✳
12	Age at 1st Pregnancy	✳	✳	*E*	*Disease*		
13	BF duration^*^	✳	✳	28	Hx of previous diseases	✳	✳
14	Dominant BF side	✳	✳	29	Type of IGM treatment	✳	
15	Hx of Inf	✳	✳	30	Recent IGM side	✳	
16	Hx of Inf treatment	✳	✳	31	Specific comments	✳	✳

* BF * Breastfeeding, *HRT* hormone replacement therapy, *Hx* History, *Inf* infertility

^*^The sum of all periods of lactation, ^**^ considered in participants who opted for the measurements

During the project, feedback indicated participant reluctance regarding neck, waist, and hip circumference measurements. Consequently, these measurements were made optional, resulting in substantial missing data for these variables (ranging from one-third to half of the cases, as shown in Table 1). This limitation was deemed acceptable, and participants were not excluded solely because of missing anthropometric data.

The variables were categorized as necessary, important, or useful (Table 2). The exclusion criteria were as follows: participants missing data for more than one necessary variable, more than two important variables, or more than three useful variables. Since participants in both groups were selected based on their willingness and ability to complete dietary assessments, missing diet-related data were minimal.

**Table 2:** Classification of Nondiet variables according to importance in the study

Class			
Important	Necessary	Useful	Other^*^
Variables			
Patient age	Age at menarche	City of birth	Neck circumference
Gravidity	Abortion Hx	Province of birth	Waist circumference
Parity	Age at 1st Pregnancy	City of residence	Hip circumference
Weight	Hx of Inf	Province of residence	
Height	Duration of OCP use	Education	
Hx of previous diseases	Last OCP use	Job	
BF duration	Dominant BF side	Hx of HRT	
Hx of OCP use	Hx of Inf treatment	Duration of HRT	
		Last HRT	
		Type of IGM treatment	
		Recent IGM side	

*BF* Breastfeeding, *HRT* hormone replacement therapy, *Hx* History, *Inf* infertility

^*^Variables that were only considered in participants who opted for the measurements and are missing in a large portion of participants

Overall, envisaging all the data, including general and system-related data (such as participant codes, durations of interviews, participant and physician names, etc.), 430,572 data points were created and finalized for this project.

## Discussion

We conducted a project to develop a comprehensive dataset of dietary information for IGM patients and a control group. The methods used for data collection and clearance, and the features of the dataset are presented in this manuscript.

Dietary composition is one of the most important modifiable environmental factors affecting human health. As a result, the relationships between diet and various diseases, including specific categories such as malignancies and inflammatory conditions, have been extensively studied. Although the association between breast cancer and nutrition has been widely explored, benign breast disease (BBD) has also been investigated in this context. Aghababayan et al. compared dietary inflammatory index scores [17] and phytochemical indices [18] between women with and without BBD and reported a slightly positive association for the former and a negative association for the latter. Similarly, Tiznobeyk et al. [19] reported an inverse relationship between a healthy dietary pattern and BBD. More recently, Rastad et al. [20] examined multiple aspects of dietary habits in BBD. However, none of these studies included idiopathic granulomatous mastitis (IGM). From a broader perspective, the link between diet and inflammatory conditions has been well established through numerous studies [9, 10, 21, 22].

The inflammatory nature of IGM, its probable link to autoimmunity, and the triggering effect of milk stasis in a subset of women collectively suggest a more substantial association between IGM and dietary factors. Nevertheless, this detail remains obscure due to the scarcity of studies on this subject. Yurdacan et al. [12] compared serum IgG antibodies against 54 food allergens in 32 IGM patients and their matched controls. They reported significantly greater intolerance to lentils and curry in IGM and concluded that these factors may contribute to IGM pathogenesis. Among many other potential contributing factors, Zeng et al. [4] compared the use of spicy food in 594 cases and controls and reported no significant differences between the groups. Deng et al. [11] implemented a case management model on 152 patients with IGM to evaluate the factors affecting disease recurrence. One of the recommendations in the model was to abstain from eating exciting food, consisting of spicy foods and other considerations. While they reported that approximately 14.5% of the patients used these items before they affected them, they did not examine whether adherence to the food protocol affected recurrence rates.

There has been a link between the gut microbiota and breast inflammation in animal studies [23, 24], but a probable link with IGM has been considered in only one study. Dai et al. [13] compared the characteristics of gut microbiota metabolites (namely, short-chain fatty acids) in stool samples from 35 IGM patients and 26 healthy women. They revealed significant differences between the two groups, suggesting an association between gut microbiota dysbiosis and IGM. Although dietary concerns were not directly considered, they imply such a relationship given the known association between the type of food intake and the gut microbiota [25, 26].

These preliminary findings highlight a profound knowledge gap, warranting systematic investigation into possible associations between dietary factors and IGM. Such an investigation depends on the existence of detailed data on the dietary intake of IGM patients and comparisons with a control group selected with precise criteria. While such comprehensive data were not available until now, our project has provided this essential understructure. We present a dietary dataset encompassing 147 food items, quantifying consumption patterns, in a substantial number of IGM patients and a rigorously selected control group. This unique resource sets the ground for effective investigations into the associations between diet and IGM. In addition, related factors, including BMI components and reproductive and hormonal variables, are included in the dataset, enabling the consideration of confounding factors.

To date, physicians managing IGM patients have either avoided dietary instructions or provided recommendations based on personal opinions. On the basis of the present dataset, authentic research may yield validated dietary advice for IGM patients instead of the current speculative approaches.

The present dataset represents the first dedicated dietary resource for IGM research. Key strengths of this study include the consistent data collection process, the use of a validated tool, and its clinical relevance.

Although we planned to attract international collaboration, we have not yet succeeded in this endeavor. However, our next planned projects will assess the associations between IGM and the dietary inflammatory index, vitamin D, and B-group vitamins, and the healthy eating index. This dataset also opens avenues for additional research, and international collaborations would pave the way toward evidence-based dietary guidelines for IGM management.

## Limitation

This study had some limitations. As with most research involving food frequency questionnaires, the potential for recall bias exists. Our choice of controls (companions of patients and outpatient visitors of selected clinics) may not fully eliminate the risk of selection bias. While community-based random sampling could theoretically reduce selection bias, this approach would have been logistically challenging in a multicenter study of this scale. Thus, the selected control group represented a practical and appropriate comparison population, according to our rationale explained in the Methods. Additionally, our population is limited to Iran, which may affect the generalizability of the findings to other populations.

## Conclusion

This study presents the process of developing a large dietary regimen for IGM patients and a rigorously selected control group. It includes quantitative data approximately 147 food items collected through a validated tool and other related variables collected via standardized protocols. The data set sets the background for research on the associations between IGM and dietary details and will allow the definition of dietary guidelines for IGM patients.

## Supplementary Information

Below is the link to the electronic supplementary material.

Supplementary Material 1.

## Data Availability

The datasets analyzed during the current study are available from the corresponding author on reasonable request.

## References

[1] Kessler E, Wolloch Y. Granulomatous mastitis: a lesion clinically simulating carcinoma. Am J Clin Pathol. 1972;58(6):642–6. 4674439 10.1093/ajcp/58.6.642

[2] Metanat S, et al. Global distribution of idiopathic granulomatous mastitis: a scoping review: IGM global distribution. Arch Breast Cancer. 2022;9(3–SI):261–71.

[3] Krawczyk N, et al. Idiopathic granulomatous mastitis as a benign condition mimicking inflammatory breast cancer: current Status, knowledge gaps and rationale for the GRAMAREG study (EUBREAST-15). Cancers (Basel). 2024;16(19):3387. 39410007 10.3390/cancers16193387 PMC11476029

[4] Zeng Y, et al. Predisposing factors for granulomatous lobular mastitis: A Case–Control study. Int J Womens Health. 2023;15:1063–75. 37795195 10.2147/IJWH.S414054 PMC10547110

[5] Alipour S, et al. Predisposing factors for idiopathic granulomatous mastitis: a large prospective multicentric study. Iran J Public Health. 2025;54(3):645–53. 40330183 10.18502/ijph.v54i3.18258 PMC12051815

[6] Sadaf Alipour MT, Aziminezhadan P, Boloorinejad P, Orouji A, Farbod M, Nafissi N, Madani F, Saberi A, Mazinani A. Maryam Daneshpazhooh, Ramesh Omranipour, Reza Hosseinpour, Paniz FamilAmir, Maryam Sarkardeh, Mahsa Tavakol, Abdolali Assarian, Bita Eslami, from hypothesis to robust evidence in a nationwide multicenter case–control study on 1490 women: milk stasis and idiopathic granulomatous mastitis. Sci Rep. 2025 (in press). 10.1038/s41598-025-10784-7 PMC12569042 41152262

[7] Omranipour R, Mohammadi SF, Samimi P. Idiopathic granulomatous lobular mastitis - report of 43 cases from iran; introducing a preliminary clinical practice guideline. Breast Care (Basel). 2013;8(6):439–43. 24550752 10.1159/000357320 PMC3919426

[8] Dilaveri C et al. Idiopathic granulomatous mastitis. Breast J. 2024;2024: 6693720. 38304866 10.1155/2024/6693720 PMC10834090

[9] Hart MJ, et al. Dietary patterns and associations with biomarkers of inflammation in adults: a systematic review of observational studies. Nutr J. 2021;20(1):24. 33712009 10.1186/s12937-021-00674-9 PMC7955619

[10] Koelman L, Egea Rodrigues C, Aleksandrova K. Effects of dietary patterns on biomarkers of inflammation and immune responses: a systematic review and Meta-Analysis of randomized controlled trials. Adv Nutr. 2022;13(1):101–15. 34607347 10.1093/advances/nmab086 PMC8803482

[11] Deng Y, et al. A case management model for patients with granulomatous mastitis: a prospective study. BMC Womens Health. 2022;22(1):143. 35501850 10.1186/s12905-022-01726-w PMC9063211

[12] Yurdacan M, et al. Food intolerance and allergy: do they have an etiological role in idiopathic granulomatous mastitis? J Clin Med. 2025;14(3):940. 39941611 10.3390/jcm14030940 PMC11818162

[13] Dai Q, et al. Characteristics of metabolites analysis for patients with granulomatous lobular mastitis. Front Cell Infect Microbiol. 2025;15:1514315. 40557323 10.3389/fcimb.2025.1514315 PMC12186243

[14] Esfahani FH, et al. Reproducibility and relative validity of food group intake in a food frequency questionnaire developed for the Tehran lipid and glucose study. J Epidemiol. 2010;20(2):150–8. 20154450 10.2188/jea.JE20090083 PMC3900814

[15] Ghaffarpour M, Houshiar-Rad A, Kianfar H. The manual for household measures, cooking yields factors and edible portion of foods. Tehran: Nashre Olume Keshavarzy. 1999;7(213):42–58.

[16] Kalantari N. G.M., Appendixes of National report of the comprehensive study on household food consumption patterns and nutritional status of I.R.Iran, 2001–2003. 2005, nutrition research Group, National nutrition and food technology research Institute, Shaheed Beheshti University of Medical Sciences, Ministry of Health: Tehran, I.R. Iran. p. 151–91.

[17] Aghababayan S, et al. Higher dietary inflammatory index scores are associated with increased odds of benign breast diseases in a Case–Control study. J Inflamm Res. 2020;13:61–9. 32104043 10.2147/JIR.S232157 PMC7008174

[18] Aghababayan S, et al. Dietary phytochemical index and benign breast diseases: A Case–Control study. Nutr Cancer. 2020;72(6):1067–73. 31475586 10.1080/01635581.2019.1658795

[19] Tiznobeyk Z, et al. Dietary patterns and benign breast diseases: a case–control study. Br J Nutr. 2016;116(2):353–9. 27198589 10.1017/S0007114516002002

[20] Rastad H, et al. Association of weight Indicators, dietary Habits, and physical activity with common benign breast diseases: an observational Case–Control study. Top Clin Nutr. 2024;39(2):165–74.

[21] Minihane AM, et al. Low-grade inflammation, diet composition and health: current research evidence and its translation. Br J Nutr. 2015;114(7):999–1012. 26228057 10.1017/S0007114515002093 PMC4579563

[22] Ricker MA, Haas WC. Anti-Inflammatory diet in clinical practice: A review. Nutr Clin Pract. 2017;32(3):318–25. 28350517 10.1177/0884533617700353

[23] Shively CA, et al. Consumption of mediterranean versus Western diet leads to distinct mammary gland Microbiome populations. Cell Rep. 2018;25(1):47–e563. 30282037 10.1016/j.celrep.2018.08.078 PMC6338220

[24] Hu X, et al. Targeting gut microbiota as a possible therapy for mastitis. Eur J Clin Microbiol Infect Dis. 2019;38(8):1409–23. 31079312 10.1007/s10096-019-03549-4

[25] Zmora N, Suez J, Elinav E. You are what you eat: diet, health and the gut microbiota. Nat Rev Gastroenterol Hepatol. 2019;16(1):35–56. 30262901 10.1038/s41575-018-0061-2

[26] Fu J, et al. Dietary fiber intake and gut microbiota in human health. Microorganisms. 2022;10(12):2507. 36557760 10.3390/microorganisms10122507 PMC9787832

