# Supplementary material for: Development of a comprehensive dietary dataset for idiopathic granulomatous mastitis (IGM) patients and matched controls: protocol, implementation, and future directions for nutrition-based research
Source: BMC Res Notes. 2025 Oct 22;18:441. doi: 10.1186/s13104-025-07507-6 (PMC12541929; doi:10.1186/s13104-025-07507-6)
Supplement: Supplementary file 1 — Supplementary Material 1. [file 13104_2025_7507_MOESM1_ESM.pdf]

**Feed Frequency Questionnaire**

Date: - / - / -                                                                                                    Patient Code: ----

| Code | Foods | Amounts | Average use last year | | | | Description |
|---|---|---|---|---|---|---|---|
| | | | Day | Week | Month | Year | |
| 1 | Bread (Lavash) | | | | | | |
| 2 | Bread (Barbari) | | | | | | |
| 3 | Bread (Sangak) | | | | | | |
| 4 | Bread (Taftoon) | | | | | | |
| 5 | Bread (Baguette) | | | | | | |
| 6 | Wholemeal bread | | | | | | |
| 7 | Bread (Other) | | | | | | |
| 8 | Cooked rice | | | | | | |
| 9 | Baked pasta | | | | | | |
| 10 | Vermicelli | | | | | | |
| 11 | Noodle | | | | | | |
| 12 | Wheat flour | | | | | | |
| 13 | Cooked barley | | | | | | |
| 14 | Baked oatmeal | | | | | | |
| 15 | Lentils | | | | | | |
| 16 | Beans | | | | | | |
| 17 | Pea | | | | | | |
| 18 | Broad bean | | | | | | |
| 19 | Soy | | | | | | |
| 20 | Mung bean | | | | | | |
| 21 | Cotyledon | | | | | | |
| 22 | Beef or Veal | | | | | | |
| 23 | Lamb meat | | | | | | |
| 24 | Minced meat | | | | | | |
| 25 | Chicken with skin | | | | | | |
| 26 | Chicken without skin | | | | | | |
| 27 | Fish (except canned tuna) | | | | | | |
| 28 | Canned tuna | | | | | | |
| 29 | Hamburger | | | | | | |
| 30 | Sausage | | | | | | |
| 31 | Cold cuts | | | | | | |
| 32 | Purtenance | | | | | | |
| 33 | Tripe and abomasum | | | | | | |
| 34 | Tongue | | | | | | |
| 35 | Sheep's brain | | | | | | |
| 36 | Sheep's head | | | | | | |
| 37 | Sheep's trotters | | | | | | |
| 38 | Egg | | | | | | |
| 39 | Skim milk (Less than 2.5%) | | | | | | |

| | | | | | | | |
|---|---|---|---|---|---|---|---|
| 40 | Milk (2.5 %) | | | | | | |
| 41 | Full fat milk | | | | | | |
| 42 | Chocolate milk | | | | | | |
| 43 | Milk chocolate | | | | | | |
| 44 | Strained yogurt | | | | | | |
| 45 | Low fat yogurt | | | | | | |
| 46 | Full fat yogurt | | | | | | |
| 47 | Creamy yoghurt | | | | | | |
| 48 | Cheese (except cream and Liquon cheese) | | | | | | |
| 49 | Cream and Liquon cheese | | | | | | |
| 50 | Dough | | | | | | |
| 51 | Cream | | | | | | |
| 52 | Traditional ice cream | | | | | | |
| 53 | Non-traditional ice cream | | | | | | |
| 54 | Butter | | | | | | |
| 55 | Margarine | | | | | | |
| 56 | Dried whey, Curd | | | | | | |
| 57 | Lettuce | | | | | | |
| 58 | Tomato | | | | | | |
| 59 | Cucumber | | | | | | |
| 60 | Green vegetables fines herbes | | | | | | |
| 61 | Stewed vegetables | | | | | | |
| 62 | Pumpkin | | | | | | |
| 63 | Squash | | | | | | |
| 64 | Eggplant | | | | | | |
| 65 | Celery | | | | | | |
| 66 | Potato | | | | | | |
| 67 | French fries | | | | | | |
| 68 | Green Peas | | | | | | |
| 69 | Green beans | | | | | | |
| 70 | Raw carrots | | | | | | |
| 71 | Steamed carrots | | | | | | |
| 72 | Garlic | | | | | | |
| 73 | Raw onions | | | | | | |
| 74 | Fried onion | | | | | | |
| 75 | Cabbage | | | | | | |
| 76 | Bell pepper | | | | | | |
| 77 | Raw spinach | | | | | | |
| 78 | Steamed spinach | | | | | | |
| 79 | Turnip | | | | | | |
| 80 | Mushrooms | | | | | | |
| 81 | Green pepper | | | | | | |
| 82 | Corn | | | | | | |
| 83 | Tomato paste or Ketchup | | | | | | |
| 84 | Pickle | | | | | | |
| 85 | Salty pickles | | | | | | |
| 86 | Pickles (Cucumber) | | | | | | |

| | | | | | | | |
|---|---|---|---|---|---|---|---|
| 87 | Cantaloupe | | | | | | |
| 88 | Melon | | | | | | |
| 89 | Watermelon | | | | | | |
| 90 | Pear | | | | | | |
| 91 | Apricot | | | | | | |
| 92 | Cherries | | | | | | |
| 93 | Apple | | | | | | |
| 94 | Peach | | | | | | |
| 95 | Nectarine | | | | | | |
| 96 | Greengage | | | | | | |
| 97 | Figs | | | | | | |
| 98 | Dried fig | | | | | | |
| 99 | Grape | | | | | | |
| 100 | Kiwi | | | | | | |
| 101 | Grapefruit | | | | | | |
| 102 | Orange | | | | | | |
| 103 | Persimmon | | | | | | |
| 104 | Tangerine | | | | | | |
| 105 | Pomegranate | | | | | | |
| 106 | Date | | | | | | |
| 107 | Plum | | | | | | |
| 108 | Sour cherry | | | | | | |
| 109 | Strawberry | | | | | | |
| 110 | Banana | | | | | | |
| 111 | Chino | | | | | | |
| 112 | Sour lemon | | | | | | |
| 113 | Grapefruit juice | | | | | | |
| 114 | Orange juice | | | | | | |
| 115 | Apple juice | | | | | | |
| 116 | cantaloupe juice | | | | | | |
| 117 | Cranberries | | | | | | |
| 118 | Pineapple | | | | | | |
| 119 | Canned pineapple | | | | | | |
| 120 | Raisins | | | | | | |
| 121 | Dew melon | | | | | | |
| 122 | Fresh berries | | | | | | |
| 123 | Dried berries | | | | | | |
| 124 | Dried peach | | | | | | |
| 125 | Dried apricot | | | | | | |
| 126 | Fruit compote | | | | | | |
| 127 | Olive | | | | | | |
| 128 | Hydrogenated oil | | | | | | |
| 129 | Liquid oil | | | | | | |
| 130 | Olive oil | | | | | | |
| 131 | Tallow | | | | | | |
| 132 | Animal oil | | | | | | |
| 133 | Mayonnaise | | | | | | |
| 134 | Peanut | | | | | | |
| 135 | Almond | | | | | | |

| | | | | | | | |
|---|---|---|---|---|---|---|---|
| 136 | Walnut | | | | | | |
| 137 | Pistachio | | | | | | |
| 138 | Hazelnut | | | | | | |
| 139 | Seeds | | | | | | |
| 140 | Biscuits | | | | | | |
| 141 | Crackers | | | | | | |
| 142 | Cake (Yazdi) | | | | | | |
| 143 | Homemade cake | | | | | | |
| 144 | Other cakes | | | | | | |
| 145 | Cookies | | | | | | |
| 146 | Pie | | | | | | |
| 147 | Tea | | | | | | |
| 148 | Sugar Loaf | | | | | | |
| 149 | Sugar | | | | | | |
| 150 | (Halva) A sweet breakfast food in Iran | | | | | | |
| 151 | Honey | | | | | | |
| 152 | Jam | | | | | | |
| 153 | Chocolate / Cocoa / Toffee | | | | | | |
| 154 | Candy | | | | | | |
| 155 | rock candy | | | | | | |
| 156 | Cheetos | | | | | | |
| 157 | Chips | | | | | | |
| 158 | Gaz (kind of sweet in Iran) | | | | | | |
| 159 | Sohan (kind of sweet in Iran) | | | | | | |
| 160 | Noghl ( kind of Sugarplum) | | | | | | |
| 161 | Caramel cream | | | | | | |
| 162 | Homemade halva | | | | | | |
| 163 | Donut | | | | | | |
| 164 | Coffee / Nescafe | | | | | | |
| 165 | Soft drinks | | | | | | |
| 166 | Industrial fruit juice | | | | | | |
| 167 | Lemon juice | | | | | | |
| 168 | Salt | | | | | | |
